# Spontaneous Conception During Ovarian Stimulation Complicated by Severe Ovarian Hyperstimulation Syndrome (OHSS) With Atypical β-Human Chorionic Gonadotropin (β-hCG) Trends: A Case Report

**DOI:** 10.7759/cureus.107732

**Published:** 2026-04-26

**Authors:** Lara Nahouli, Nathalie Chamseddine, Omar Alameddine, Ghadir Aouad, Ghina Ghazeeri

**Affiliations:** 1 Obstetrics and Gynecology, American University of Beirut Medical Center, Beirut, LBN

**Keywords:** abnormal trend, ascites, hcg levels, ohss, viable pregnancy

## Abstract

Ovarian hyperstimulation syndrome (OHSS) is a known complication of ovarian stimulation and may complicate the interpretation of β-human chorionic gonadotropin (β-hCG) in early pregnancy. Importantly, pregnancy can still occur during ovarian stimulation, and in this setting, ectopic pregnancy must be carefully excluded, given the potential for tubal dysfunction related to fluid shifts, increasing diagnostic uncertainty.

We present a 28-year-old woman who developed severe OHSS following controlled ovarian stimulation, in whom spontaneous conception occurred prior to planned intrauterine insemination. Serial β-hCG levels showed an initial plateau, likely reflecting fluid shifts and residual exogenous hCG, followed by a delayed rise, raising concern for abnormal pregnancy. However, early ultrasound confirmed a viable intrauterine pregnancy, and the patient subsequently delivered a healthy infant.

This case highlights the limitations of relying solely on β-hCG trends in OHSS and underscores the importance of integrating clinical findings and early ultrasound to guide management.

## Introduction

Human chorionic gonadotropin (hCG) is a glycoprotein hormone produced primarily by syncytiotrophoblastic cells of the placenta and serves as a key biomarker for the detection and monitoring of early pregnancy viability. While hCG is predominantly metabolized in the liver, approximately 20% is excreted unchanged in the urine [1].

Traditionally, a doubling of serum hCG levels every 48 hours was considered indicative of a viable intrauterine pregnancy. However, more recent evidence suggests that the rate of hCG increase varies depending on the initial serum concentration. Lower initial levels (<1,500 mIU/mL) are typically associated with an approximate 49% increase over 48 hours, whereas higher initial levels (>3,000 mIU/mL) may demonstrate a slower rise of approximately 33% [2]. Despite this variability, most viable intrauterine pregnancies exhibit increases exceeding these minimum thresholds. Therefore, a suboptimal rise in hCG levels over 48 hours raises concern for abnormal pregnancy, including ectopic pregnancy or early pregnancy loss.

Chung et al. demonstrated that hCG rise patterns are comparable between spontaneously conceived pregnancies and those achieved through assisted reproductive technologies, with expected increases of approximately 50% at 24 hours and 124% at 48 hours [3]. Notably, the minimal rise associated with eventual live birth was reported to be 14% at 24 hours and 30% at 48 hours [3].

Several factors may influence hCG kinetics in early pregnancy. Among these, ovarian hyperstimulation syndrome (OHSS) has been associated with atypical hCG trends. Limited studies suggest that patients with OHSS may exhibit slower-than-expected increases in hCG levels despite ultimately having viable pregnancies [4,5].

OHSS is characterized by increased vascular permeability leading to third spacing, hemoconcentration, and subsequent fluid redistribution. These dynamic changes may alter serum biomarker concentrations, including β-hCG, complicating its interpretation in early pregnancy. In this context, distinguishing between abnormal pregnancy and physiologic variation becomes particularly challenging. This report highlights a unique clinical scenario of spontaneous conception during ovarian stimulation complicated by severe OHSS, emphasizing the diagnostic limitations of relying solely on β-hCG trends.

Herein, we present a case of a 28-year-old woman with severe OHSS who demonstrated an initially abnormal rise in hCG despite a clinically viable intrauterine pregnancy.

## Case presentation

A 28-year-old nulliparous woman with a history of polycystic ovarian syndrome (PCOS), biliary atresia status post liver transplant at the age of one year, and lymphoma diagnosed at the age of five (treated with chemotherapy and subsequent discontinuation of immunosuppressive therapy), presented with severe abdominal pain, bloating, and shortness of breath. The patient had been undergoing controlled ovarian stimulation in preparation for intrauterine insemination (IUI) following two years of primary infertility at an outside institution. She received daily low-dose (75 IU) of human menopausal gonadotropin (HMG) injections for eight days, followed by an hCG trigger. As this was an IUI stimulation protocol rather than in vitro fertilization (IVF), a gonadotropin-releasing hormone (GnRH) antagonist was not used, and estradiol levels were not routinely monitored. Notably, she reported unprotected sexual intercourse three days prior to administration of the hCG trigger.

On the day of the planned procedure, she developed abdominal pain unresponsive to analgesics. Bedside ultrasound demonstrated moderate pelvic free fluid and bilaterally enlarged ovaries. OHSS was suspected; therefore, the procedure was aborted, and the patient was discharged home on conservative management. Six days after the trigger, she presented to the emergency department with worsening abdominal distension, decreased oral intake, severe abdominal pain, and dyspnea on exertion.

On presentation, she was tachycardic (heart rate 115 beats/min). Physical examination revealed a tense, distended abdomen with diffuse tenderness and shifting dullness. Laboratory evaluation demonstrated hemoconcentration with hemoglobin of 17.1 g/dL (reference: 12-16 g/dL), hematocrit of 51% (reference: 36-46%), and leukocytosis (18,300/cu.mm; reference: 4,000-11,000/cu.mm). Additional findings included hyponatremia (130 mEq/L; reference: 135-145 mEq/L). Liver function tests were within normal limits.

She received daily low-dose (75 IU) of HMG injections for eight days, followed by an hCG trigger. As this was an IUI stimulation protocol rather than IVF, a GnRH antagonist was not used, and estradiol levels were not routinely monitored as part of the protocol.

Baseline β-hCG level was 22.5 mIU/mL, consistent with recent administration of exogenous hCG. Given the recent administration of exogenous hCG trigger, early β-hCG measurements likely reflected residual circulating hormone. Based on prior pharmacokinetic data, exogenous hCG may remain detectable for up to 10-14 days, with a gradual decline [6]. Therefore, the initial plateau and slight decline observed in this case may represent washout kinetics rather than true endogenous production.

**Figure 1:**
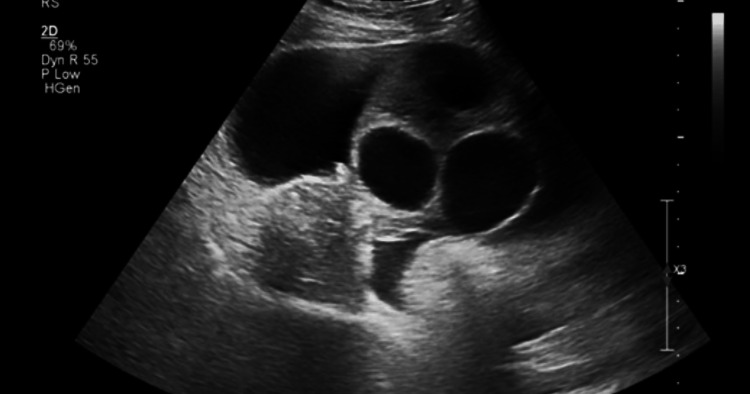
Transvaginal ultrasound showing bilaterally enlarged ovaries with multiple cystic follicles and moderate pelvic ascites.

Transvaginal ultrasound revealed bilaterally enlarged ovaries with multiple cystic follicles and moderate ascites. The largest ovarian diameter measured approximately 12 cm, and the ascites was amenable to ultrasound-guided drainage (Figure 1). Chest X-ray showed no pleural effusion.

The patient was diagnosed with severe OHSS and admitted for management. According to widely accepted clinical classification criteria, this presentation is consistent with severe OHSS, given the presence of significant ascites, hemoconcentration (hematocrit >45%), oliguria, and rapid weight gain [7]. She was started on prophylactic anticoagulation, intravenous hydration, and cabergoline. Urine output and daily weight were closely monitored.

Forty-eight hours later, β-hCG was 21.9 mIU/mL. This pattern is suggestive of exogenous hCG washout followed by emerging endogenous production. On day 1 post-admission, she gained 4 kg and developed oliguria (<0.5 mL/kg/hour), prompting abdominal paracentesis with pigtail catheter placement by the interventional radiology team. Abdominal fluid was drained slowly for symptomatic relief, with 500 mL to be drained every four hours or sooner, depending on the patient’s clinical status.

Despite intervention, the patient’s condition continued to deteriorate, with worsening abdominal distension, progressive weight gain, dyspnea, and leukocytosis (24,000/cu.mm). Pregnancy was initially considered unlikely given the absence of IUI, the attribution of early β-hCG levels to recent exogenous hCG administration, and the initial decline in β-hCG values. Given the lack of clinical and objective improvement, the possibility of pregnancy was reconsidered, despite the fact that IUI had not been performed.

**Figure 2:**
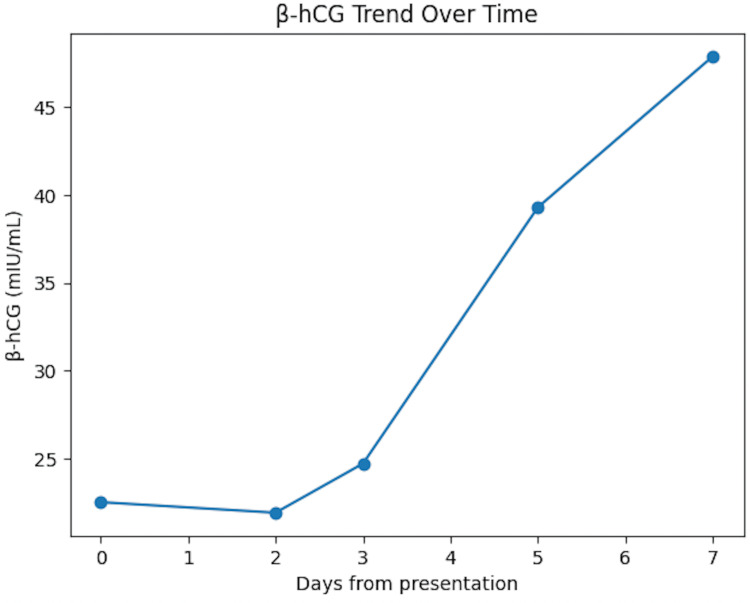
Line graph illustrating the patient’s β-hCG trend (mIU/mL) over several days following initial presentation. The x-axis represents days from presentation, and the y-axis represents serum β-hCG levels (mIU/mL). The graph demonstrates an initial decline followed by a delayed rise in β-hCG. An initial decrease of 2.67% was observed, followed by successive increases of 12.78%, 59.91%, and 21.69%, respectively. β-hCG: β-human chorionic gonadotropin

Serial β-hCG measurements demonstrated an atypical trend. The initial level was 22.5 mIU/mL, followed by a slight decrease to 21.9 mIU/mL at 48 hours. This was subsequently followed by a gradual rise to 24.7 mIU/mL after one day, 39.3 mIU/mL at 48 hours, and 47.9 mIU/mL at 96 hours (Figure 2). Based on expected elimination kinetics of exogenous hCG, a continuous decline would typically be anticipated. However, the observed shift from an initial decline to a subsequent rise suggests the transition from exogenous hormone clearance to endogenous β-hCG production.

**Figure 3:**
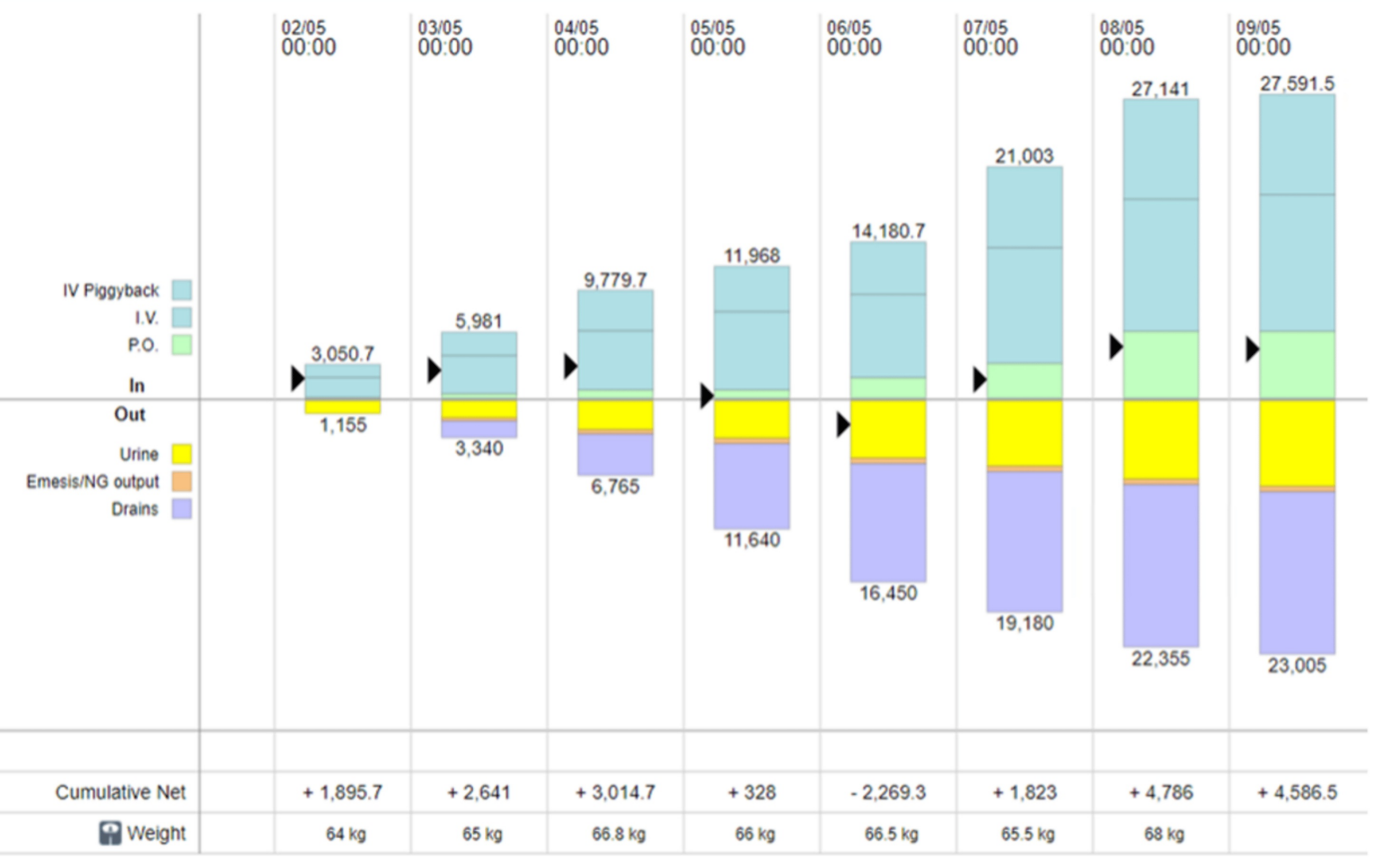
Graph showing the daily increase in weight and the daily in/out net balance. Weight is expressed in kilograms (kg), and fluid balance is expressed in milliliters per day (mL/day), illustrating the relationship between fluid shifts and clinical progression.

Cabergoline was discontinued due to a lack of clinical improvement and the transition to albumin-based management. In addition, given the possibility of an early pregnancy, continuation was carefully reconsidered, as the routine use of cabergoline in this context remains a matter of clinical judgment when not clearly indicated. The intensive care and nephrology teams were consulted, and the patient was initiated on scheduled albumin therapy (20 g every six hours) as some guidelines suggest in cases of third spacing with intravascular depletion. Fluid management, including hydration and drainage, was adjusted on a daily basis according to her clinical status. Overall, she experienced a total weight gain of 9 kg from the time of diagnosis of OHSS (Figure 3).

The patient was discharged home after nine days with the pigtail catheter in place following clinical improvement. She was provided with strict instructions regarding warning signs of ectopic pregnancy, and β-hCG levels were monitored on an outpatient basis. An intrauterine pregnancy was confirmed at four weeks and five days of gestation, with fetal cardiac activity detected at six weeks and five days (Table 1). The pigtail catheter was removed at eight weeks of gestation after complete resolution of the ascites.

**Table 1:** Chronological summary of patient events and β-hCG levels. β-hCG: β-human chorionic gonadotropin; OHSS: ovarian hyperstimulation syndrome

Day	Event
Day -3	Unprotected intercourse
Day 0	hCG trigger administered
Day +6	Presentation to the emergency department, diagnosis of OHSS
Day +6	β-hCG 22.5 mIU/mL
Day +8	β-hCG 21.9 mIU/mL
Day +9	β-hCG 24.7 mIU/mL
Day +11	β-hCG 39.3 mIU/mL
Day +13	β-hCG 47.9 mIU/mL
Day +33	Intrauterine pregnancy
Day +47	Positive fetal cardiac activity

The remainder of the pregnancy was complicated by pyelonephritis during the third trimester, for which she received intravenous antibiotics. She subsequently presented in labor at 37 weeks and six days of gestation and delivered a healthy female infant via spontaneous vaginal delivery. The intrapartum and postpartum courses were uncomplicated.

## Discussion

Ovulation induction is a cornerstone in the management of female infertility; however, it may occasionally result in an exaggerated ovarian response known as OHSS [7]. OHSS is a potentially life-threatening condition characterized by increased vascular permeability, leading to fluid extravasation, intravascular volume depletion, and hemoconcentration.

The interpretation of β-hCG trends in early pregnancy is an essential component in assessing pregnancy viability. However, in the context of OHSS, this interpretation becomes more complex. Several studies have demonstrated that the expected rise in β-hCG may be altered in patients with OHSS, potentially leading to diagnostic uncertainty [4,5].

Early literature on this topic is limited. A small observational study published in 2004 suggested that abnormal β-hCG rise in OHSS should not be solely used to diagnose ectopic or nonviable pregnancy [4]. More recent evidence supports this observation. A case-control study involving 82 patients with moderate to severe OHSS demonstrated significantly lower serum β-hCG levels and a slower doubling time compared to controls, with an approximately 18% slower rise (p=0.014) [8]. Compared to previously reported cases, including a case by Benor et al. demonstrating suboptimal β-hCG rise despite a viable intrauterine pregnancy [9], our patient demonstrated a more pronounced initial decline followed by a delayed rise, likely reflecting the combined effects of exogenous hCG washout and OHSS-related fluid shifts. In contrast, most prior reports describe a slowed but continuously rising β-hCG pattern.

Choux et al. further described altered β-hCG kinetics in patients with severe OHSS, while noting that obstetric and neonatal outcomes were not adversely affected [10]. The altered β-hCG kinetics observed in OHSS may be explained by dynamic intravascular volume changes. During the acute phase, increased vascular permeability leads to third spacing and hemoconcentration. As fluid shifts back into the intravascular compartment during recovery or following interventions such as paracentesis and albumin administration, hemodilution may occur. These fluctuations can transiently alter measured β-hCG concentrations without reflecting true trophoblastic activity. The atypical β-hCG rise in this case appeared temporally associated with changes in fluid status and clinical interventions, supporting the hypothesis that intravascular dilution and redistribution contributed to the observed hormonal trends. Choux et al. reported that hCG levels exhibited a slower increase prior to paracentesis, likely due to hemodilution, whereas a greater rise was observed afterward as the dilutional effect diminished [10].

In our case, the initial β-hCG level was attributed to recent administration of exogenous hCG. It is well established that exogenous hCG may remain detectable for up to 14 days following administration [6]. The subsequent decline in β-hCG levels may reflect redistribution effects associated with fluid shifts. A key diagnostic challenge in this case was distinguishing between exogenous and endogenous β-hCG contributions. The initial decline likely reflects residual exogenous hormone, while the subsequent rise suggests endogenous production. Failure to recognize this transition may lead to misdiagnosis.

As the patient’s clinical status continued to deteriorate, pregnancy was reconsidered, particularly given her history of unprotected sexual intercourse three days prior to administration of the hCG trigger. This is biologically plausible, as Ferreira-Poblete reported that human sperm have approximately a 5% probability of surviving beyond 4.4 days [11].

Key clinical pitfalls in this setting include misinterpreting atypical β-hCG trends as indicative of nonviable pregnancy, overlooking the possibility of spontaneous conception during stimulation cycles, and failing to account for residual exogenous hCG. Importantly, abnormal β-hCG patterns in OHSS may mimic ectopic pregnancy; however, biochemical trends alone are insufficient for diagnosis, and ultrasound remains essential for accurate localization.

This case highlights the limitations of relying solely on β-hCG trends in patients with OHSS. A comprehensive approach incorporating clinical findings, serial measurements, and imaging is essential to avoid misdiagnosis and inappropriate management.

## Conclusions

This case highlights a unique and clinically important scenario of spontaneous conception during ovarian stimulation complicated by severe OHSS, resulting in atypical β-hCG kinetics. Clinicians should be cautious when interpreting early β-hCG trends in this setting, particularly in the presence of recent exogenous hCG administration and significant fluid shifts. A structured approach incorporating clinical status, serial measurements, and early ultrasound is essential to avoid misdiagnosis. Awareness of this diagnostic pitfall can prevent unnecessary interventions and improve patient outcomes.
